# Chimeric peptide constructs comprising linear B-cell epitopes: application to the serodiagnosis of infectious diseases

**DOI:** 10.1038/srep13364

**Published:** 2015-08-21

**Authors:** Yudong Lu, Zhong Li, Huan Teng, Hongke Xu, Songnan Qi, Jian’an He, Dayong Gu, Qijun Chen, Hongwei Ma

**Affiliations:** 1 Nano-Bio-Chem Centre, Suzhou Institute of Nano-Tech and Nano-Bionics, Chinese Academy of Sciences, Suzhou, 215123, P. R. China; 2 Graduate University of Chinese Academy of Sciences, Beijing, 100049, P. R. China; 3 Suzhou SJ Biomaterials Co. Ltd. Suzhou, 215123, P. R. China; 4 Central Laboratory of Health Quarantine, Shenzhen International Travel Health Care Center, Shenzhen Entry-exit Inspection and Quarantine Bureau, Shenzhen, 518033, P. R. China; 5 Key Laboratory of Zoonosis, Jilin University, Changchun, 130062, P. R. China

## Abstract

Linear B-cell epitopes are ideal biomarkers for the serodiagnosis of infectious diseases. However, the long-predicted diagnostic value of epitopes has not been realized. Here, we demonstrated a method, diagnostic epitopes in four steps (DEIFS), that delivers a combination of epitopes for the serodiagnosis of infectious diseases with a high success rate. Using DEIFS for malaria, we identified 6 epitopes from 8 peptides and combined them into 3 chimeric peptide constructs. Along with 4 other peptides, we developed a rapid diagnostic test (RDT), which is able to differentiate *Plasmodium falciparum* (*P. falciparum*) from *Plasmodium vivax* (*P. vivax*) infections with 95.6% overall sensitivity and 99.1% overall specificity. In addition to applications in diagnosis, DEIFS could also be used in the diagnosis of virus and bacterium infections, discovery of vaccine candidates, evaluation of vaccine potency, and study of disease progression.

For diseases of unknown causes (e.g., autoimmune diseases), proteomics^1^,^2^,^3^,^4^ are often involved in searching for biomarkers, and random peptide/peptoid microarrays have also achieved occasional success^2^,^5^,^6^. Conversely, for infectious diseases caused by a parasite, bacteria or virus it is clear where to look for biomarkers: either antigens from pathogens or antibodies in serum. Although parasitological detection (e.g., thick smears or PCR) is still the gold standard for the diagnosis of infectious disease, the high technical requirements of the operators and the time-consuming process make it unsuitable for large-scale disease surveillance. The protein biomarker-based rapid diagnostic test (RDT) is more commonly used. The diagnosis of *P. falciparum* malaria was typically based on the detection of a *P. falciparum*-specific antigen, namely, the histidine rich protein II (*P. falciparum* HRPII)^7^,^8^ with matched monoclonal antibodies (mAbs; Fig. 1a). This traditional strategy relies heavily on the discovery of species-specific antigens, which could be a long and costly journey full of uncertainties^1^,^8^,^9^,^10^. Furthermore, both genetic and immunogenic variation could cause false negatives for such antigen-based immunoassays. For example, approximately 5% of *P. falciparum* does not naturally express the *P. falciparum* HRPII gene^8^,^11^. We identified that 7.6% of patients actually carry HRPII antibodies (Extended Data Fig. 1), and they are also negative for HRPII detection, which leads to a total of 12.6% intrinsic false-negative results. Another diagnostic biomarker of malaria, lactate dehydrogenase, was also found to have neutralizing antibodies (Extended Data Fig. 1), implying that all protein biomarkers face such intrinsic false-negative problems due to the existence of neutralizing antibodies.

Antibodies in serum are ideal biomarkers for diagnosis to avoid the false-negative problem described above. Epitopes, as the antibody recognition region of the antigen, could be used for antibody detection. The diagnostic value has been long predicted but not realized. Many epitopes have been identified from extensive studies on malaria^12^,^13^,^14^,^15^, yet no epitope-based diagnostic tools are in use. We attribute this to four problems: (1) technical difficulties in large-scale seroscreening of peptide microarrays;^16^ (2) limited numbers of linear epitopes^17^ available as biomarkers from a single protein; (3) the complexity of antibodies in serum;^18^ and (4) immune diversity causing contradiction between sensitivity and specificity^19^. All these four obstacles can be overcome by using DEIFS, a standardized procedure that is not only general but also practical in finding epitope combinations of diagnostic value. Figure 1b summarizes the four steps of DEFIS and the rest of the paper will describe the method in great detail.

## Results

### Two-round seroscreening and three-mode analysis

**Figure 2:**
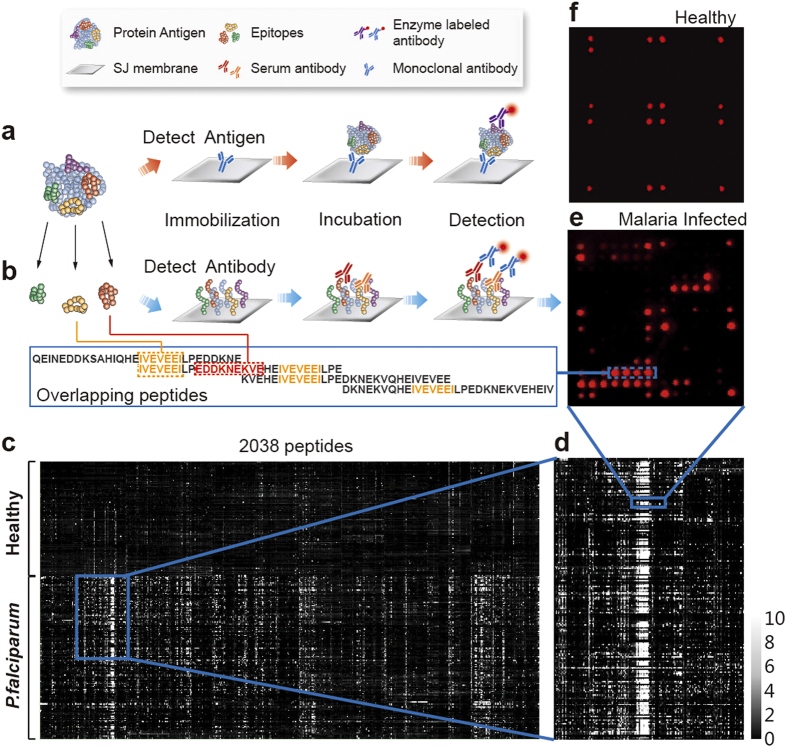
Biomarkers for serodiagnosis. Both antigen and antibodies can be the target of detection. (**a**) *P. falciparum* specific antigen can be detected by sandwiched immunoassay to indicate infection. (**b**) Epitopes are immunodominant regions of antigen protein, which can interact with antibodies. Antigens were divided into overlapped peptides (30 amino acids in length with 15 amino acids overlapped), which were printed on iPDMS membranes to form microarrays. (**c**) A heat map of 304 serums (179 *P. falciparum* infected, 125 healthy) to 2038 peptides was obtained by directly converting SNR to grayscale. (**d**) In large-scale seroscreening, specific antibodies-epitope interactions resulted in high signal intensity region on the heat map. (**e**) Representative result from *P. falciparum* infected serum. (**f**) Representative result from healthy serum.

For the first step of DEIFS, 38 *P. falciparum* proteins (Fig. 2b and Extended Data Table 1) were selected and divided into 2038 overlapped peptides for candidate library construction. These 2038 overlapped peptides were printed on iPDMS membrane to form a microarray chip for the second step of **DEIFS**, a two-round seroscreening, which was conducted for a training group (125 healthy and 289 *P. falciparum*-infected serum). The iPDMS membrane provides a near “zero” background for serological assays, even without blocking treatment (Fig. 2e,f). With this unique feature, the data acquisition and analysis were simple^20^ (Extended Data Fig. 2): chemiluminescence intensity was captured by a CCD camera for each dot of the microarray, which was then converted to the signal to noise ratio (SNR). These original data could be used to conduct the following bioinformatics analysis. Thus, we solved the first problem: technical difficulties in the large-scale seroscreening of peptide microarrays. The solution for the second problem was using multiple proteins, as demonstrated below, by which one could obtain enough epitopes for diagnosis.

**Figure 3:**
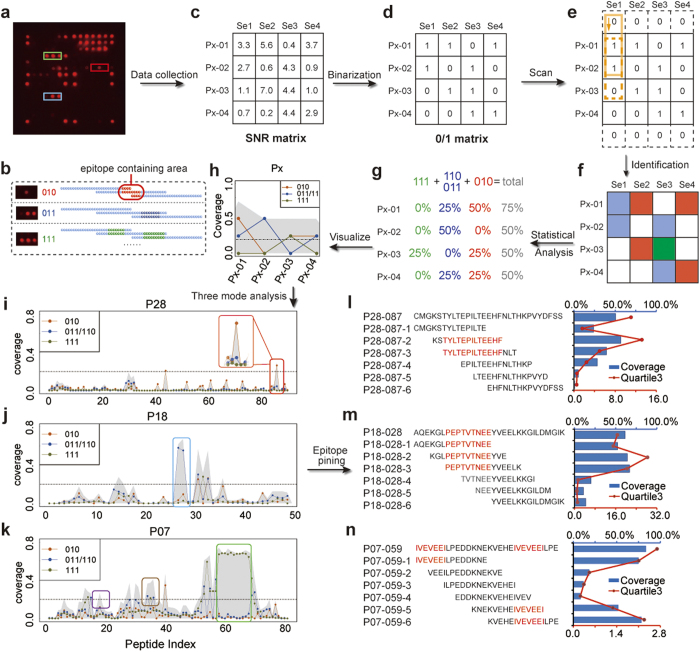
Three-mode analysis and two round screening strategy for ECPs identification. (**a**) For instance, 010, 011 and 111 modes were, respectively, indicated with red, blue and green boxes. (**b**) A peptide with the 010 mode or 011/110 mode indicated a single epitope in the 30 aa peptide. A peptide with the 111 mode indicated that the protein contains more than a single epitope in the 30 aa peptide. (**c**) For each serum, each peptide obtained an SNR value after seroscreening (as an example, protein Px was resolved into 4 peptides and reacted with 4 serums, which are Se1, Se2, Se3 and Se4). The SNR matrix of protein Px was converted to (**d**) 1/0 matrix at the beginning of the three-mode analysis. (**e**) After modes identification, the 1/0 matrix was converted to (**f**) a mode type matrix, which was then (**g**) statistically analyzed to (**h**) visualize treatment. The line chart with dots represents coverage of 3 different modes, which were 010 (h, red line), 011/110 (h, blue line) and 111 (**h**, green line). The area chart (**h**, gray area) represents the total coverage of all 3 modes. The three-mode analysis revealed epitope containing peptides (ECPs). The three-mode analysis of P28 (**i**), P18 (**j**) and P07 (**k**) are typical instances revealing ECPs from three different modes. Selected ECPs were subjected to a second-round screening (15/12 aa) for epitope pinning. Different modes showed different epitope locations: type 010 in the middle (**l**), type 011/110 in the common parts of two adjacent 30 aa peptides (**m**) and type 111 represented a series of epitopes repeatedly located in 3 consecutive peptides (**n**). The identified epitope sequences were selected to make chimeric peptides.

The first round of seroscreening used peptides of 30 amino acid (aa) in length with 15 aa overlapping (abbreviated as 30/15 aa thereafter). A heat map was obtained by converting the resulting SNR value to grayscale (Fig. 2b–d). Significance Analysis of Microarray (SAM), which is widely used in DNA microarray analysis, performed poorly for the peptide microarrays when used directly on the large-scale original data of the peptide microarrays (Extended Data Table 3). Here we introduced a “three-mode analysis” method (Fig. 3), which facilitated the identification of epitope containing peptides (ECPs).

First, for the ease of algorithm design, SNR values larger than the cutoff (SNR ≥ 2) were converted to 1, otherwise to 0. As a result, the SNR matrix (serum *vs.* peptides) was converted to a 1/0 matrix (Fig. 3c,d). Second, three successive peptides along the protein sequence were studied as one unit. The first analysis unit was in solid frame Px-01 to Px-03 (Fig. 3e). The second analysis unit was in dashed frame, Px-02 to Px-04, and so on. Theoretically, there are six combinations (i.e., modes) for three successive values, namely 000, 001/100, 010, 011/110, 101, and 111. However, these six modes are not analytically equivalent. We calculated the percentage of serums belonging to each mode, as shown in the line/area chart (Fig. 3h). We only focused on the coverage of three unique modes for epitope identification: namely, the 010, 011 and 111 modes, indicated with red, blue and green boxes in Fig. 3a. The coverage of the 000, 001/100 and 101 modes was designated as 0 because the 000 mode had no contribution to epitope identification and the 001/100 and 010 modes could be represented by the 010 or 011/110 modes. Each of the three modes represents a form of epitope location (Fig. 3b). We can easily identify ECPs through the line/area chart of the three-mode analysis. We analyzed all 38 proteins by three mode analysis (Extended Data Fig. 3) because an ECP is defined as a peptide with high SNR to the majority of infected serum samples. The cutoff value for coverage was arbitrarily selected as 20%, as shown by the dashed lines in Fig. 3i–k. Peaks above the dashed line indicated the location of the ECPs. A peptide with the 010 mode or 011/110 mode indicated a single epitope in the 30 aa peptide (Fig. 3l,m). A peptide with the 111 mode indicated that the protein contains a repeat sequence of ECPs (Fig. 3n). The shadow area behind the line chart indicates the total coverage of the three modes. For diagnosis, ECPs with high coverage of the 010 or 011/110 modes and similar coverage of the shadow area are preferred, indicating a single epitope of high sensitivity. Moreover, the relationship between the line chart and area chart (i.e., the gray shadow) reflected the complexity of the epitope composition: if an ECP has a low coverage of each of the three modes but a high coverage in total (i.e., area chart), we can infer that the multiple epitopes were contained in this ECP. Although those epitopes contributed little to the diagnosis due to insufficient sensitivity, they might give some hint to the research of pathogenesis: why do patients show different immune responses when suffering from the same pathogen invasion? Using the three-mode analysis, only 153 out of 2038 peptides were identified as ECPs. This 7.4% rate is an average of 38 *P. falciparum* proteins (Extended Data Table 3), which is in agreement with the previously reported 2% value of B-cell linear epitopes^17^. Only these 153 peptides were further subjected to the second round seroscreening, using 15 aa with 12 aa overlapped peptides to pin the location of the epitope sequences, which confirmed the reliability of our “three-mode analysis” method for identifying epitopes. For example (shown in Fig. 3l–n), P28-87 is a peptide with a high coverage of mode 010. The second round screening revealed the “TYLTEPILTEEHF” sequence as the epitope sequence. Similarly, P18-028 had a high coverage of mode 110/011, and its epitope was located in the common sequence “‘PEPTVTNEE”. P7-059 had a high coverage of mode 111 and was one of the peptides contained in the highly repeated sequence of “KNEKVEHEIVEVEEILPE” for P7. We concluded that the “IVEVEEI” sequence is essential for antibody recognition, which was supported by Michael *et al*. using phage display^12^.

### Optimization of the epitope combination and binarization/digitization

**Figure 4:**
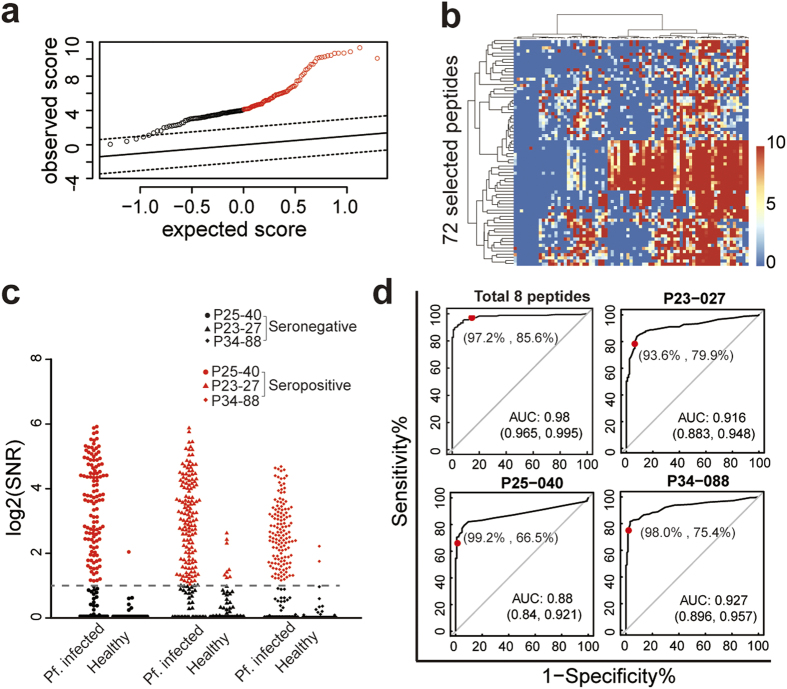
Optimization of the epitope combination. (**a**) SAM plot of 153 selected peptides from *P. falciparum* infected and healthy samples. A total of 72 peptides with significantly different SNR in infected and healthy samples are shown in red circles. (**b**) The heat map of these 72 peptides was clustered, and 8 peptides were selected from each cluster. (**c**) Violin plot of 3 selected peptides (P25-040, P23-027, and P34-088). (**d**) ROC curve of each of the 3 peptides and all 8 peptides. Although these peptides have high specificity (99.2%, 93.6%, and 98.0%), none has satisfactory sensitivity (66.5%, 79.9%, and 75.4%).

After reducing 2038 peptides to 153 ECPs by the three-mode analysis (Extended Data Table 3), SAM succeeded in extracting peptides that show different responsive rates for different serum subgroups (Fig. 4a): a total of 72 ECPs were selected as highly responsive peptides in *P. falciparum*-positive samples and were clustered (Fig. 4b). ECPs with the highest coverage of positive serum from each cluster group were selected. For further optimization, we calculated the total coverage of these ECPs. Eight ECPs (Table 1) were finally identified as diagnostic candidates. The 8 selected ECPs from multiple proteins performed poorly based on the traditional SNR cutoff value (Fig. 4c,d and Extended Data Figs 4 and 5 and Table 4), where SNR ≥ 2 indicated a positive result. None of the 8 ECPs can provide a satisfactory sensitivity (>90%) of diagnosis. Only 2 of the 8 ECPs could achieve 100% specificity, which was attributed to mimotopes or molecular mimics due to the complexity of the antibodies in the serum. If we used the traditional strategy of multiplexing, i.e., any one of the 8 ECPs being positive indicates that the serum is positive^21^, one would find an increased sensitivity of 97.2% but a poor specificity of 86.4%. We believe that this contradiction between sensitivity and specificity has hindered the realization of the long-predicted diagnostic value of epitopes. A binarization/digitization strategy was developed to enable the 8 selected ECPs to achieve diagnostic function, a solution for the third and fourth problems.

First, we defined a universal binary cutoff value of SNR = 2 as the indication of being responsive (Extended Data Fig. 2c–e). For more than 10,000 microarrays assayed, the mean SNR of blank dots (i.e., negative control dots printed with buffer) was 0.5 ± 0.3, so the value of (blank + 3 std) is approximately 1.4. We chose 2 as a strict and more conservative cutoff. This stable and near zero background value is critical for the binarization treatment: if a peptide has SNR < 2, we assign *D*_*i*_ = 0; if SNR ≥ 2, *D*_*i*_ = 1, where *D* represents the digital diagnosis and *i* represents a peptide. We do not judge whether this response is due to specific interaction. Indeed, an SNR of 2 could be due to low concentrations of specific antibodies (i.e., specific interaction) but is more likely due to nonspecific interactions because there are as many as 10^5^ different antibodies in human serum (i.e., the third problem). Instead, we rely on probability to determine if the response is from specific interactions.

**Figure 5:**
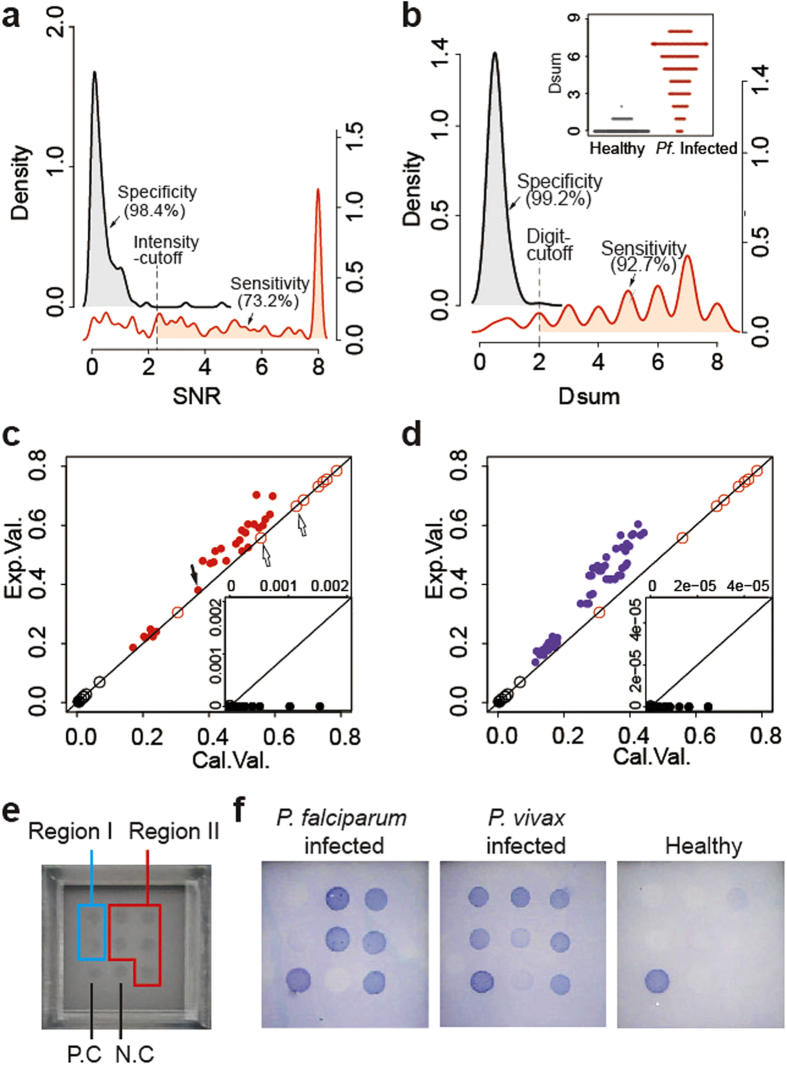
Binarization/digitalization strategy and RDT validation (**a**) Under the single index mode with intensity-cutoff at SNR = 2.3, P34-88 had a 73.2% sensitivity and 98.4% specificity. The inserted figure gives an overlapped view of two curves (**b**). Under binarization/digitalization and setting the binary-cutoff (SNR) and digit-cutoff (n) both at 2, the 8-peptide resulted in 92.7% sensitivity and 99.2% specificity. (**c**) Correlation of 28 two-peptide combinations for the *P. falciparum* infected training group. The Exp. value was the number obtained from experiments. For digit-cutoff n = 1, the Cal. value was the same as the Exp. value: these open dots are located on the diagonal line. For n = 2, the Cal. value (the solid red dots indicated by the filled black arrow) is the product of any two Exp. values (the open red dots indicated by the two open black arrows). (**d**) Correlation of 56 three-peptide combinations for the training group. For n = 3, the Cal. value (the solid purple dots) is the product of any three Exp. values. (**e**) For RDT (a 3 × 3 array layout): positive control, negative control and 7 other peptides were arrayed as Region I and II. (**f**) Any two of the five dots in Region II showing positive results indicated malaria infection; otherwise, the indication was negative for malaria. For malaria infected serum, two dots in Region I indicated positive *Pv.* infection. No dots indicated *P. falciparum* infection. A single dot could not be certainly interpreted, and a third party test is required.

Second, we further assign:
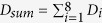
, where *D*_*sum*_ is a variable similar to the role of SNR and n is the digit-cutoff value for the digitized diagnostic microarray. For example, if the digit-cutoff n = 2, *D*_*sum*_ = 0 and 1 indicate healthy (i.e., negative for *P. falciparum*-specific antibodies), and *D*_*sum*_ ≥ 2 indicates *P. falciparum* infection (i.e., positive for *P. falciparum*-specific antibodies). A surprising improvement was observed when we set the digit-cutoff to n = 2; both the sensitivity and specificity increased from below 73.2% to 92.7% and from 98.4% to 99.2%, respectively (Fig. 5a,b).

The mechanism behind the binarization/digitization treatment is as follows: we use binarization to mark responsive dots as 1 and nonresponsive dots as 0, which allows the use of *D*_*sum*_ to indicate the number of responsive dots. Then, we use digit cutoff n to confirm positives and to eliminate false positives by the simultaneous occurrence of multiple responsive dots (*D*_*sum*_ ≥ n). When we set the digit-cutoff n = 2, any two responsive peptides indicate a positive result. The 8 peptides thus gave 28 combinations (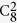). There are two sensitivity values for each of these 28 combinations: (i) the calculated value and (ii) the experimental value (Fig. 5c and Extended Data Fig. 6). If we assume that the event of a peptide being an epitope is an independent event, the probability of two peptides simultaneously being epitopes is the simple product of the two individual probabilities, i.e., the calculated sensitivity for the combination of Pep5 and Pep7 is the value of the X-axis of the dot specified by the filled black arrow in Fig. 5c, 0.659 × 0.553 = 0.364. Alternatively, we can obtain the sensitivity from the experimental data, which is the value of the Y-axis of the same dot, 0.380. The sum of 28 combinations thus gave a sensitivity as high as 98.4% while maintaining a near 100% specificity. Increasing the digit cutoff n to 3 demonstrates how simple probability can make a great difference (Fig. 5d).

### Validation with the test group

A test group containing 244 *P. falciparum* serums and 1043 control serums was subjected to the above 8 peptide combination. Under *D*_*sum*_ = 2, we achieved satisfactory sensitivity and specificity at 98.6% and 98.0%, respectively. Given that a rapid diagnostic test would be better for malaria, we changed the chemiluminescence to 3,3′,5,5′-tetramethylbenzidine (TMB) colorization, which removes the need for an instrument. One more critical improvement is the use of chimeric epitopes constructs. For 6 ECPs with identified epitopes, we created 3 chimeric epitope constructs that have improved sensitivity (Table 1). Thus, only 5 peptides were printed on the microarray, making the usage easier (Fig. 5e,f). We also achieved satisfactory sensitivity and specificity at 94.7% and 99.1%, respectively. The missed 5% was found in the early stage of infection and could be detected with IgM (data not shown).

It was beyond our expectation that the ECPs of *P. falciparum* origin would also work for *P. vivax* infection. Although *P. vivax*-infected serum showed different sensitivity (Table 1), the 5 ECPs combination gave a 91.4% sensitivity (under *D*_*sum*_ = 2). BLAST results indicated that *P. falciparum* and *P. vivax* shared homology for those 5 peptides and for many of the other peptides that we screened (Extended Data Table 5). To differentiate *P. falciparum* and *P. vivax* infection, we applied the **DEIFS** to an additional two proteins with low homology, namely, *P. falciparum* and *P. vivax* CSP (Extended Data Table 1), and obtained two additional peptides, *P. vivax* CSP-9 and *P. falciparum* CSP-24. A combination of 7 ECPs was found to have an overall sensitivity of 95.6%, an overall specificity of 99.1%, a *P. falciparum* sensitivity of 94.7% and a *P. vivax* sensitivity of 96.7%.

## Discussion

As demonstrated above, **DEIFS** solved all four identified problems that prevent the application of epitopes in diagnosis. With the two-round screening strategy, only 2038 peptides of 30/15 aa s and 978 peptides of 15/12 aa overlaps were synthesized to complete epitope mapping of 38 candidate proteins, whereas the traditional screening strategy required at least 10240 peptides of 15/12 aa overlaps. The two-round screening strategy not only saves workload but also saves nearly 70.5% of the cost. Theoretically, in silico-predicated method could reduce the cost of DEFIS by reducing the number of peptides used. However, Bergmann-Leitner reported in a recent paper that the accuracy of prediction algorithms relies heavily on a “training” process using data from related proteins^22^. Detailed comparison between silico-prediction and DEFIS method will be reported elsewhere.

Immune diversity is the major hurdle that prevents the realization of the long-predicted diagnostic value of epitopes. The heat map (Fig. 2c) is a visual representation of immune/epitope diversity. This epitope diversity is due to subject diversity: for the 289 sera tested, we did not find any two sera that had the same response/epitope pattern, nor did we find any single epitope response to all 289 serum samples. Pep4 (Table 1) is the best performing ECP with seroprevalence of only 78.2%, which means that nearly a quarter of the malaria-infected population lacks antibodies that recognize the epitope in Pep4. The same antigen may result in different epitopes due to inter-subject variations of the immune system. A three-mode analysis strategy was developed and successfully applied to reveal epitopes of diagnostic value. Furthermore, we applied a binarization/digitization strategy to overcome the contradiction between the sensitivity and specificity. The binarization strategy is not just a mathematical trick but a new perspective for diagnostic interpretation. The traditional strategy uses SNR to judge the concentration differences of antibodies/antigens between positive and negative samples. Our binarization strategy uses D_sum_ to judge the various differences in antibodies between patients and healthy people. The latter is more insensitive to individual fluctuations, which leads to a more robust strategy.

Microscopy is still regarded as the gold standard for the diagnosis of all *Plasmodium* species^23^. Unfortunately, it requires skilled professionals who are lacking in the epidemic areas of these diseases^8^,^24^. Compared with RDT based on *P. falciparum* HRPII, this 7 peptide digitized microarray is free from false negatives due to *P. falciparum* HRPII gene deletion. Compared with PCR, the peptide microarray is cheaper and could be more informative if a high-content diagnostic microarray could be developed, which is currently ongoing in our laboratory. We have also succeeded in expanding this **DEIFS** strategy to other infectious diseases, such as tuberculosis (bacterium) and hand-foot-and-mouth disease (HFMD, virus), as long as the immune system responds to the infection by producing antibodies. Although a large-scale linear B-cell epitope screening is the prerequisite for such digitized diagnostic microarray development and requires a certain cost, the overall cost could be shared because the microarray may also find a use in the discovery of vaccine candidates, the evaluation of vaccine potency, and the study of disease progression. There are still many infectious diseases lacking proper diagnostic tools, especially neglected tropical diseases, because the identification process involves genomics, protein engineering and many other disciplines^25^ and is not economically affordable. The standardized DEIFS method only costs $100,000 and three months to develop an RDT, an estimation based on EV71 virus (10 proteins), which causes HFMD. Thus, **DEIFS** is readily applicable to other infectious diseases, especially to those neglected tropical diseases that cause significant economic burden and humanitarian crises in less developed countries, by producing a chip that contains 1000 thousand peptides and is able to screen 200 pathogens in one test.

## Methods

### Serum

A total number of 924 malaria infected serum and 1257 healthy serum were used in this study.289 (179 + 110) samples of *P.falciparum* infected serum, 176 samples of *P.vivax* infected serum were collected from southwest area of China, which were confirmed by the microscope method; 214 (125 + 89) negative samples of healthy serum were collected from local hospitals. Those samples were used as the training group for seroscreening. 244 samples of *P.falciparum* infected serum, 215 samples of *P.vivax* infected serum, 1043 negative samples of healthy serum were obtained from Institute of Malaria Control in Yunnan province, China, which were confirmed by the microscope method. Those samples were used as the testing group for seroscreening (Extended data Table 2). Informed consent was obtained from all the subjects.

All malaria infected serum samples were collected after onset. Most of the *P.falciparum* infected serum were collected at ring stage, a few at gametophyte stage. *P.vivax* infected serum samples were collected at erythrocytic stage (merozoites, ring, trophozoite and schizont stage). Randomized, double-blind, parallel-controlled trials was conducted for test group study.

### Reagents

iPDMS membrane^26^ (15 × 15 mm2) was obtained from SJ Biomaterials (Suzhou, China). 1-ethyl-3-(3-dimethylaminopropyl) carbodiimide (EDC) and N-hydroxysuccinimide (NHS) were purchased from Medpep (Shanghai, China). Peptide (30 amino acid in length) was synthesized by GL Biochem (shanghai, China). Human IgG (H-IgG) was purchased from DGCS-Bio (Beijing, China). Horseradish peroxidase-labeled goat anti-human IgG (HRP-IgG) was obtained from ZSGB-Bio (Beijing, China). Peroxidase Conjugate Stabilizer/Diluent and Chemiluminescence substrate (SuperSignal ELISA Femto Maximum Sensitivity Substrate) were purchased from Thermo Fischer (USA). Tetramethylbenzidine (TMB) chromogenic reagent were purchased from Nanjing Jiancheng Bioengineering Institute, China. Commercial malaria test product: BinaxNow® Malaria Test 25 test kit^27^ (reorder number # : 660-000) were purchased from Alere (Shanghai) Medical Sales Co., Ltd; Wondfo® One Step Malaia P.f pan Test 100 test kit^28^ were purchased from Wondfo Biotech Co., Ltd (Guangzhou).

### Peptide microarray

Microarray was prepared in a 100,000 grade clean room. Peptides were first dissolved with 30% acetonitrile solution (v/v, in Milli-Q water) to 1 mg mL^−1^ stock solution and then diluted into 200 μg mL-1 with printing buffer (0.3M PB, 0.2% Glycerin, 0.01% Triton and 1.5% Mannitol) for further printing. iPDMS membranes were first activated with 0.1M EDC and 0.1 M NHS mixtures for 30 min and then rinsed with Milli-Q water and used for printing immediately. For the training group, we used a homemade reaction chamber to conduct a double side screening for repeated experiment (Extended data Fig. 2a). Microarray was prepared using contact printer Smart 48 (Capitalbio, Beijing, China) with about 0.6 nL printing solution for each sample. All the peptide samples were printed in single to form 7 × 7 × 4 array, each sub-array has positive control with H-IgG at the concentration of 100 μg mL^−1^ and negative control with printing buffer (Extended data Fig. 2b).For the test group, microarray was prepared using non-contact printer sciFLEXARRAYER S1 (Scienion Co., Berlin, Germany) with 200 drop of 0.4 nL printing solution for each peptide in triplicates. H-IgG was also spotted as the positive control at a concentration of 100 μg mL^−1^ and printing buffer was spotted as the negative control.

### Test procedure

Serum was first diluted 1 : 200 with serum-dilution buffer (1% bovine serum albumin, 1% Casein, 0.5% Sucrose, 0.2% Polyvinylpyrrolidone, 0.5% Tween20 in 0.01M Phosphate Buffered Saline, pH = 7.4) and 200 μL was added into each peptide microarray, incubated for 30 min on the shaker (150 rpm, 22 ^°^C). Microarray incubated with serum-dilution buffer was conducted as negative control. The microarray was then rinsed for 3 times with washing buffer and incubated with 200 μL of 1 mg/mL HRP-IgG diluted 1:20000 in Peroxidase Conjugate Stabilizer/Diluent for another 30 min on the shaker (150 rpm, 22 ^°^C), followed by the same washing steps as described above. 15 μL of Chemiluminescence substrate was added onto the microarray and the Images were taken at a wavelength of 635 nm using LAS4000 imaging system (GE, USA). Data analysis were conducted according to the workflow of Extended data Fig. 2d–h. For the RDT experiment, 100 μL serum sample of 1 : 10 dilution were incubated for 15 min on the shaker (150 rpm, 22 ^°^C), rinsed for 3 times with washing buffer and incubated with 100 μL of 0.8 mg/mL HRP-IgG diluted 1 : 8000 in Peroxidase Conjugate Stabilizer/Diluent for 15 min on the shaker (150 rpm, 22 ^°^C), followed by the same washing step. 100 μL TMB chromogenic reagent was added onto the microarray and stand for 3 min to read the results.

### Statistical analysis

The statistical analysis were all computed by R version 3.0.0 (R Foundation for Statistical computing, Vienna, Austria, ISBN 3-900051-07-0, URL http://www.R-project.org). Significant analysis of microarray (SAM) is performed using “samr” package. Cluster analysis and heat map are performed using “pheatmap” package. Violin plot is performed using “beeswarm” package. Receiver Operator Characteristic curve (ROCC) is performed using “OptimalCutpoints” package. Sensitivity and specificity were calculated according the following formulas: Sensitivity = True positive/(True positive + False negative) × 100%; Specificity = True negatives/(False positives + True negatives) × 100%. The positive predictive value (PPV) is defined as PPV = True positive/(True positive + False positive) × 100%; The negative predictive value (NPV) is defined as NPV = True negative/(True negative + False negative) × 100%. Evaluations of statistically significant differences between the tests were based on sensitivity, specificity, negative and positive predictive values. The area under the curve (AUC) was used to validate diagnostic application of the 8 ECPs (Extended data Table 6). Agreement between categorized indices was assessed by Cohen’s kappa coefficient (poor: 0 < κ < 0.4; fair: 0.4 < κ < 0.6; good: 0.6 < κ < 0.8; excellent: 0.8 < κ < 1). (Extended data Table 7)

### Ethics statement

This study was approved by the ethics committee of Shenzhen International Travel Health Care Center. All the experiments described here were performed in accordance with the approved guidelines.

## Additional Information

**How to cite this article**: Lu, Y. *et al*. Chimeric peptide constructs comprising linear B-cell epitopes: application to the serodiagnosis of infectious diseases. *Sci. Rep.* **5**, 13364; doi: 10.1038/srep13364 (2015).

## Supplementary Material

Supplementary Information

## Figures and Tables

**Figure 1:**
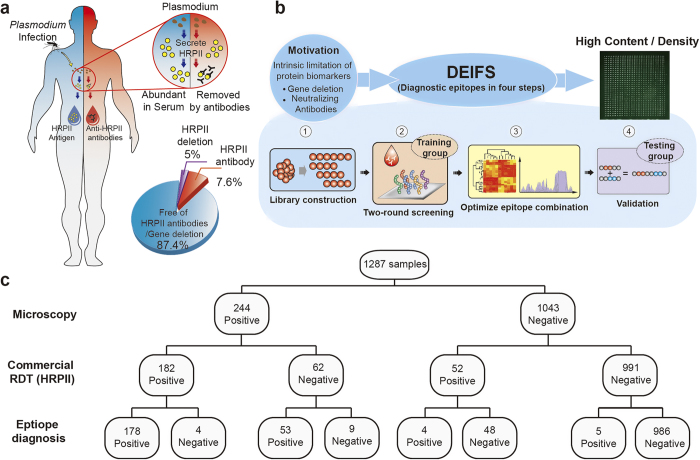
Brief illustration of finding diagnostic epitopes in four steps (DEIFS). Motivated by the intrinsic limitations of protein biomarkers (**a**), epitope based diagnoses were proposed and realized by DEIFS (**b**): 1. Library construction, protein candidates were selected and translated to 30/15 aa overlapped peptide library; 2.Two-round screening, 30/15 aa overlapped peptide library was screened by training group serum and was narrowed by three-mode analysis. Selected peptides were subjected to 15/12 aa overlapped second round screening for epitope pinning; 3. Optimization of epitopes combination. Peptides were further narrowed by SAM and Cluster algorithm and were optimized for diagnosis by the D_sum_ principle; 4. Validation, chimeric peptides were created from diagnostic ECPs for rapid diagnostic testing. The whole process could be performed in three months and could shift between high content and high density modes according to the need. Figure 2, 3, 4, 5 will give more details of each of the four steps. (**c**) Performance of the epitope diagnosis based on DEIFS. Sensitivity and specificity at 94.7% and 99.1%, respectively, were achieved.

**Table 1:**
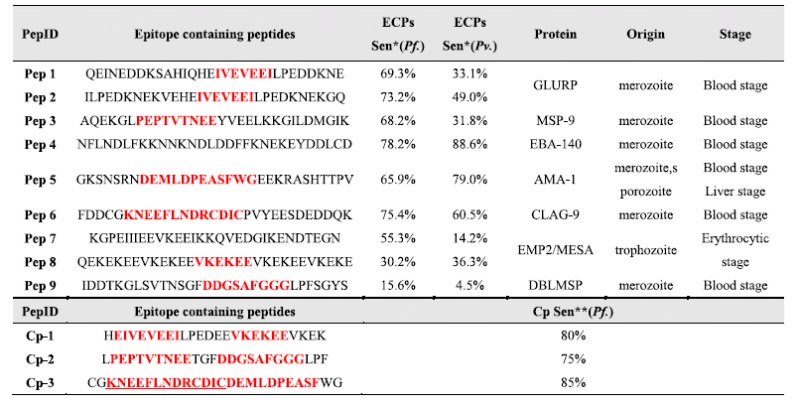
Diagnostic epitopes and their chimera.

Pep 1: Q_857_∼E_886_ from protein P7; Pep 2: I_917_∼Q_946_ from protein P7; Pep 3: A_406_∼K_435_ from protein P18; Pep 4: N_391_∼D_420_ from protein P23; Pep 5: G_586_∼V_615_ from protein P25; Pep 6: F_1306_∼K_1335_ from protein P34; Pep 7: K_586_∼N_615_ from protein P35; Pep 8: Q_912_∼E_941_ from protein P35; Pep 9: I_106_∼S_135_ from protein P6. Pep1∼Pep8 are selected as diagnostic peptides, Pep 9 is used to enhance the performance of Cp-2.Cp-1: Pep 1/Pep 2 + Pep 8; Cp-2: Pep 3 + Pep 9; Cp-3: Pep 5 + Pep 6, epitope sequences are highlighted in red. ∗ECPs sen.: Sensitivity of epitope containing peptides. ∗ ∗Cp sen.: Sensitivity of chimeric peptide constructs.
