# Supplementary material for: Chimeric peptide constructs comprising linear B-cell epitopes: application to the serodiagnosis of infectious diseases
Source: Sci Rep. 2015 Aug 21;5:13364. doi: 10.1038/srep13364 (PMC4543967; doi:10.1038/srep13364)
Supplement: Supplementary Information [file srep13364-s1.pdf]

Chimeric peptide constructs comprising linear B-cell epitopes: application to the serodiagnosis of infectious diseases

Yudong Lu[1,2†], Zhong Li[1†], Huan Teng[3], Hongke Xu[1], Songnan Qi[3], Jian'an He[4], ©Dayong Gu[4], Qijun Chen[5*], Hongwei Ma[1*]

[1]Nano-Bio-Chem Centre, Suzhou Institute of Nano-Tech and Nano-Bionics, Chinese Academy of Sciences, Suzhou, 215123, P. R. China. [2]Graduate University of Chinese Academy of Sciences, Beijing, 100049, P. R. China. [3]Suzhou SJ Biomaterials Co. Ltd. Suzhou, 215123, P. R. China. [4]Central Laboratory of Health Quarantine, Shenzhen International Travel Health Care Center, Shenzhen Entry-exit Inspection and Quarantine Bureau, Shenzhen, 518033, P. R. China. [5]Key Laboratory of Zoonosis, Jilin University, Changchun, 130062, P. R. China.

[†]*These authors contributed equally to this work.*

**E-mail: hwma2008@sinano.ac.cn., cqj@jlu.edu.cn*

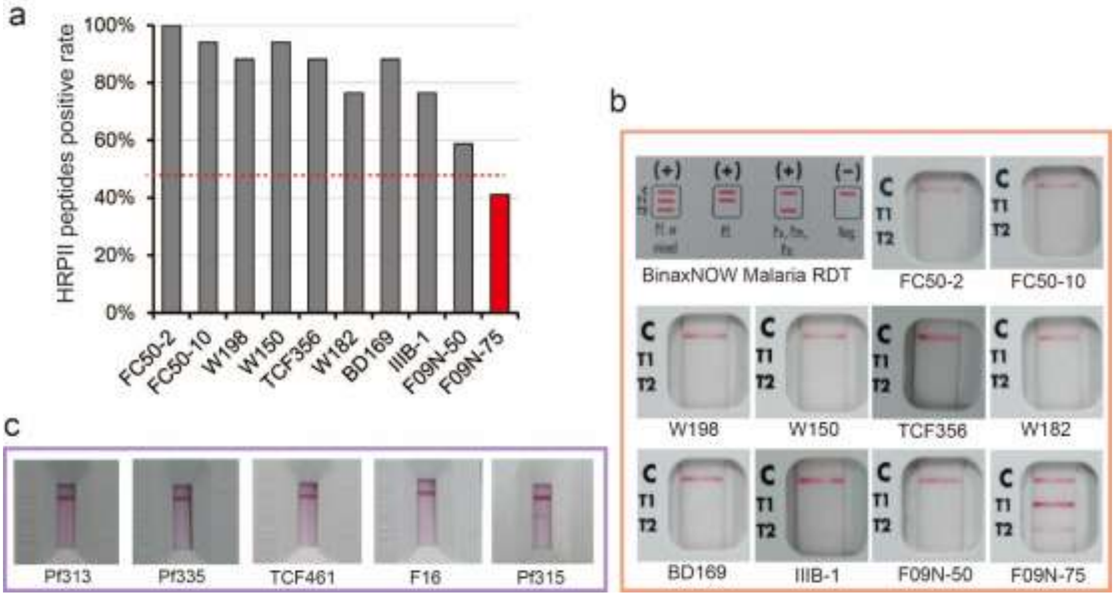

Extended Data Figure 1 The HRPII protein (P01 in **Extended Data Table 1**) was divided into 18 peptides and we defined "HRPII peptides positive rate" as the percentage of these 18 peptides with SNR ≥ 2 response from seroscreening. For example, serum FC50-2 had 18 peptides with SNR ≥ 2 response so its "HRPII peptides positive rate" was 100%. 22 out of 289 malaria positive serum were found to have over 50% "HRPII peptides positive rate". These serum were negative to a widely used commercial RDT (BinaxNow) which was based on HRPII detection. Only 1 serum (F09N-75) with 40% "HRPII peptides positive rate" had positive result in HRPII based RDT (a,b). Similarly, 5 malaria positive samples were found to carry anti-Lactate dehydrogenase (LDH) antibodies and showed false negative to LDH based RDT

(Wondfo) (c). These results demonstrated that proteins as biomarkers have to face the intrinsic false negative problem due to neutralizing antibodies.

Extended Data Table 1: Index of 40 proteins[1] used for epitope discovery*.

Protein ID	Protein name (*P. falciparum*)	NCBI ID	Protein ID	Protein name (*P. falciparum*)	NCBI ID
**P01**	HRPII	XP_002808743.1	**P21**	EBA-175	XP_001349207.2
**P02**	HRPIII	CAX64409.1	**P22**	EBA-181	XP_001350957.1
**P03**	MSP1	XP_001352170.1	**P23**	EBA-140	XP_001349859.1
**P04**	MSP2	XP_001349578.1	**P24**	EBA-165	XP_001351546.1
**P05**	MCP1	XP_001347552.1	**P25**	AMA-1	XP_001348015.1
**P06**	DBLMSP	XP_001347632.1	**P26**	PF332	XP_001348162.2
**P07**	GLURP	XP_001347628.1	**P27**	RAP1	XP_001348275.1
**P08**	MSP 3	XP_001347629.1	**P28**	RhopH2	XP_002808967.1
**P09**	MSP 4	XP_001349580.1	**P29**	RhopH3	XP_001351928.1
**P10**	MSP 5	XP_001349579.1	**P30**	CLAG2	XP_001349709.1
**P11**	MSP 6	XP_001347630.1	**P31**	CLAG3.1	XP_001351100.1
**P12**	MSP7.1	XP_001350074.1	**P32**	CLAG3.2	XP_001351099.1
**P13**	MSP7.2	XP_001350075.1	**P33**	CLAG9	XP_001352222.1
**P14**	MSP7.3	XP_002809050.1	**P34**	CLAG8	XP_002808744.1
**P15**	MSP7.4	XP_001350079.1	**P35**	EMP2/MESE	XP_001351567.1
**P16**	MSP7.5	XP_001350080.1	**P36**	Serine repeat antigen 5	XP_001349586.1
**P17**	MSP8	XP_001351583.1	**P37**	methionine-tRNA ligase	XP_001347624.1
**P18**	MSP9	XP_001350683.1	**P38**	Endoplasmic homolog	XP_001350620.1
**P19**	MSP10	XP_966190.1	**P39**	*P. vivax* CSP	AAA29535.1
**P20**	MSP11	XP_001347636.1	**P40**	*P. falciparum* CSP	ADF48458.1

*The 2038 peptides come from P01 to P38, no peptides gave a detectable response on a slide with only buffer and secondary antibody.

Extended Data Table 2. Information of serum.

	Quantity	Origin	Source	Usage
**Training group**	179	*P. falciparum*	South east of China	First-round screening
	110	*P. falciparum*		Second-round screening
	176	*P. vivax*		homology analysis
	125	*Healthy*	Jiangsu Province, East China	First-round screening
	89	*Healthy*	Liaoning province, North China	Second-round screening
**Testing group**	244	*P. falciparum*	Yunnan Province, South China	RDT test of *P. falciparum*
	215	*P. vivax*		RDT test of *P. vivax*
	1043	*Healthy*		RDT test of control

Data analysis

R_{dot} was the readout of H-IgG/peptide dot, **$R_{neg.}$** was the readout of negative control dot. All the data were extracted with AMIA Toolbox[2].

$R_{mean\ dot}$ was the mean value of 3 R_{dot} and **$R_{mean\ neg.}$** was the mean value of 3 R_{neg}.

Signal to noise ratio (SNR) was defined by the following equations:

For training group, **SNR** = $(R_{dot} - R_{mean\ neg.}) / R_{mean\ neg.}$

For test group, **SNR** = $(R_{mean\ dot} - R_{mean\ neg.}) / R_{mean\ neg.}$

The intensity-cutoff value for each individual peptide was calculated using the 125 negative samples with the following equation: **Intensity - cutoff** = $SNR_{mean} + 3\sigma$, where σ means standard deviation.

Coverage is defined as the percentage of the numbers of positive (negative) samples with SNR ≥ cutoff among the total numbers of positive (negative) samples.

Sensitivity is defined as the percentage of positive tests among the total number of positive samples.

Specificity is the percentage of negative tests among the total number of negative samples. The total number of positive/negative samples is the number detected by microscopy in blood slides.

100% - specificity is equivalent to false positive fraction.

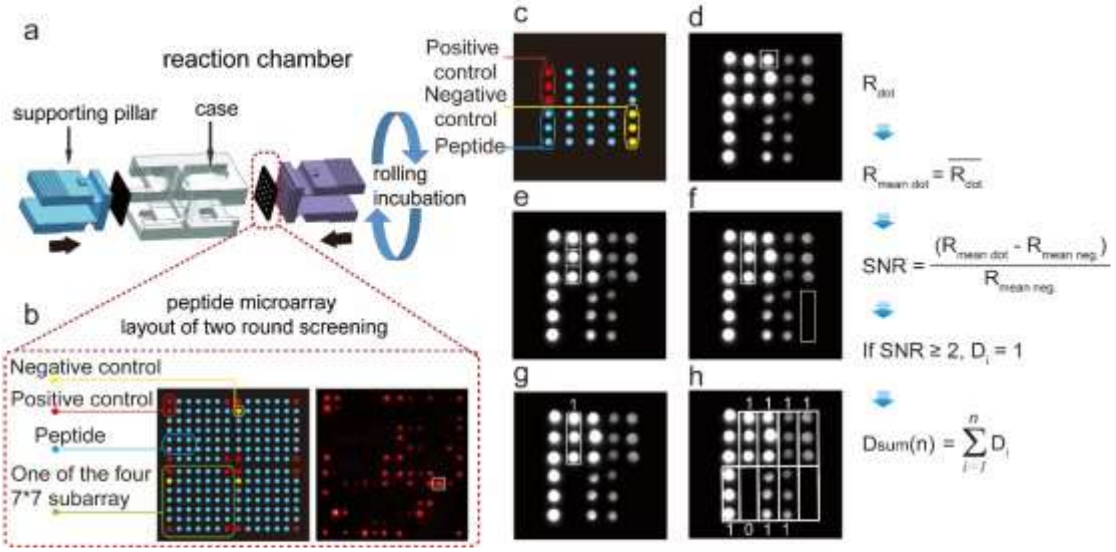

Extended Data Figure 2 Peptide microarray layout and **illustration for the workflow of data analysis.** (a) The illustration of double-side reaction chamber. Microarray chip were stuck on the supporting pillar and assembled with a plastic case to form a reaction chamber. This chamber then conducted a rolling incubation processes by a rotation incubator for homogeneous reaction. (b) The design interprets the microarray pattern on chip for training group. The array inside the green square was one of four 7×7 subarrays. The picture shows one typical result of training group. (c) The design of microarray for test group. (d-h) Data analysis protocol. (d) R_{dot} was the readout of one dot. (e) $R_{mean\ dot}$ was the mean value of 2 repeated R_{dot} for training gourp and 3 repeated R_{dot} for test group. (f) Signal to noise ratio (SNR) was calculated by the formula of

$R_{mean\ dot}$ and $R_{mean\ neg}$. (g) if a peptide has SNR ≥ 2, we assign $D_i = 1$ or else $D_i = 0$. (h) $D_{sum}(n)$ was defined as the sum of Di from one microarray. The equation on the right side reflects the corresponding data conversion process of the right protocol.

Extended Data Table 3. Results of significance analysis of microarrays (**SAM**) [3] to all peptides v.s. selected ECPs after three-mode analysis.

Protein	length(aa)	Peptides	SAM 1	ECPs	SAM 2
**P01**	278	18	9	0	0
**P02**	248	16	7	0	0
**P03**	1720	114	33	3	2
**P04**	272	18	9	0	0
**P05**	393	26	14	4	2
**P06**	697	46	23	0	0
**P07**	1233	82	41	28	23
**P08**	354	23	10	0	0
**P09**	272	18	9	3	1
**P10**	272	18	8	1	1
**P11**	371	24	12	0	0
**P12**	459	30	16	3	0
**P13**	309	20	10	1	0
**P14**	298	19	7	1	0
**P15**	281	18	9	1	0
**P16**	380	25	12	1	0
**P17**	597	39	15	1	0
**P18**	743	49	23	6	3
**P19**	525	34	15	5	4
**P20**	405	26	10	0	0
**P21**	1502	100	42	7	2
**P22**	1567	104	30	7	3
**P23**	1210	80	37	5	3
**P24**	1388	92	14	0	0
**P25**	622	41	19	8	4
**P26**	540	35	17	4	0
**P27**	782	52	23	5	2
**P28**	1378	91	23	2	0
**P29**	897	59	15	0	0
**P30**	1440	95	11	2	0
**P31**	1417	94	24	4	0
**P32**	1416	94	26	6	2
**P33**	1340	89	18	5	1
**P34**	1394	92	17	4	2
**P35**	1434	95	46	26	15
**P36**	997	66	24	4	2
**P37**	889	59	14	3	0
**P38**	821	54	12	3	0
**Sum**	31141	2038	704	153	72

The numbers for each protein of the 38 tested *P. falciparum* proteins: **length (aa)** - the total number of aa; **Peptides:** number of 30/15 overlapping peptides derived from a candidate protein**; SAM 1:** peptides (potential diagnostic epitopes) selected by SAM analysis to all peptides from a candidate protein**; ECPs:** Number of ECPs (potential diagnostic epitopes) selected by epitope scan**; SAM 2:** peptides (potential diagnostic

epitopes) selected by SAM after epitope scan**.** Differentially responsive peptide were identified by using the *t* test procedure within SAM. The P value was adjusted, and false discovery rate (FDR) < 0.05 and fold change above 2 were selected as the cutoff criteria.

Ordinary cluster arithmetic can hardly get significant classification when dealing with large numbers of samples, peptides and complicated SNR data even supported by manual adjustment of parameters. Cluster result often reflect the relationship between serum sample and peptide marker while SAM select the different expression peptide marker between positive and negative samples which is more applicable in diagnostic biomarker discovery. The application of SAM to all peptides resulted in 704 peptides (**SAM 1**) as peptides (epitopes) of diagnostic potential. By applying our three-mode analysis, only 153 peptides (**ECPs**) were selected. Further analysis of SAM could reduce this number to 72 (**SAM 2**), a reasonable number for clustering.

With the assistance of three-mode analysis, we easily identified ECPs and understood the complexity of epitope composition. This method has important reference value for further application of peptide diagnosis and even the investigation of immunopathogenesis.

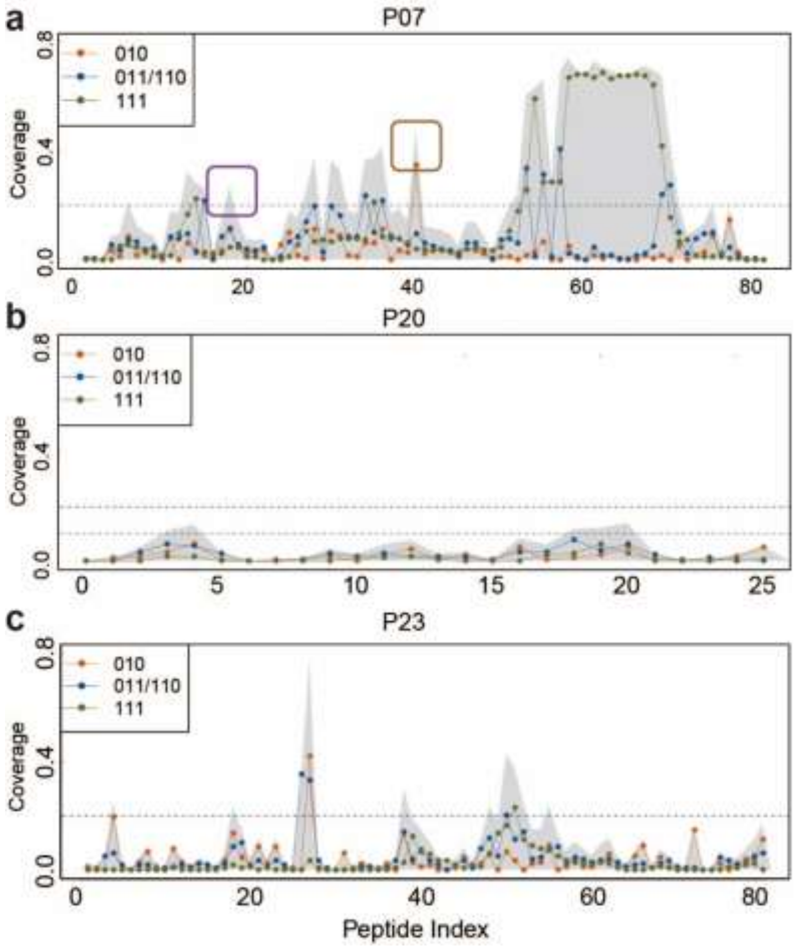

Extended Data Figure 3 The result of three-mode analysis. a-c were representive results for 38 *P. falciparum* proteins. (a) P07: abundant in epitopes; (b) P20: nearly free of epitope; and (c) P23: a medium number of epitopes.**Figure 3h, Extended Data Figure 3a-c** were constructed to visualize the statistical analysis of mode distribution for all the serum (**Figure 3g**). Horizontal axis is peptide locus and vertical axis represents the coverage value of each mode. The distribution of different mode are described as different color curves and the total coverage of all of the three modes is described as the area graph (grey) behind the curves. For example, 34.6% of serum was 010 mode for P7-40 three-mode analysis unit (brown box in **Extended Data Fig. 3a**, red line), 9.5% was 110/011 mode (blue line) and 3.9% was 111 mode (green line), the gray background stood for the sum (48.0%) of the three modes.

According to the general rule of diagnostic kit development[4], distributions of Coverage vs. SNR should be first constructed for both healthy and infected people in order to define an intensity cutoff value (**Extended Data Fig. 4**). For clarity, we further converted Frequency to Density (**Extended Data Fig. 5**). Each of the 8 ECPs could be viewed as the antigen in the traditional method (the left and middle panels of **Extended Data Fig. 4**) and Pep 6 was shown as a representative case (**Extended Data Fig. 5**). For an intensity-cutoff of SNR = 2.3, obtained was a satisfactory specificity at 98.4% (the grey area under Density curve). However, a sensitivity of 73.2% (the red area under Density curve) does not qualify Pep 6 as a diagnostic biomarker. From **Extended Data Table 4,** all of the 8 *P. falciparum* ECPs had satisfactory specificity, which reflected the fact that *P. falciparum* is a rare infection to Chinese[5]. Unfortunately, none is individually sensitive enough to be a diagnostic biomarker (i.e., > 90%). Although reset of the intensity-cutoff from SNR = 2.3 to 1 resulted in an increased sensitivity (84.4%), it was accompanied with a decreased specificity (89.6%) (**Extended Data Table 4**) Thus, we concluded that these epitopes were insufficient in sensitivity when used in the single index mode.

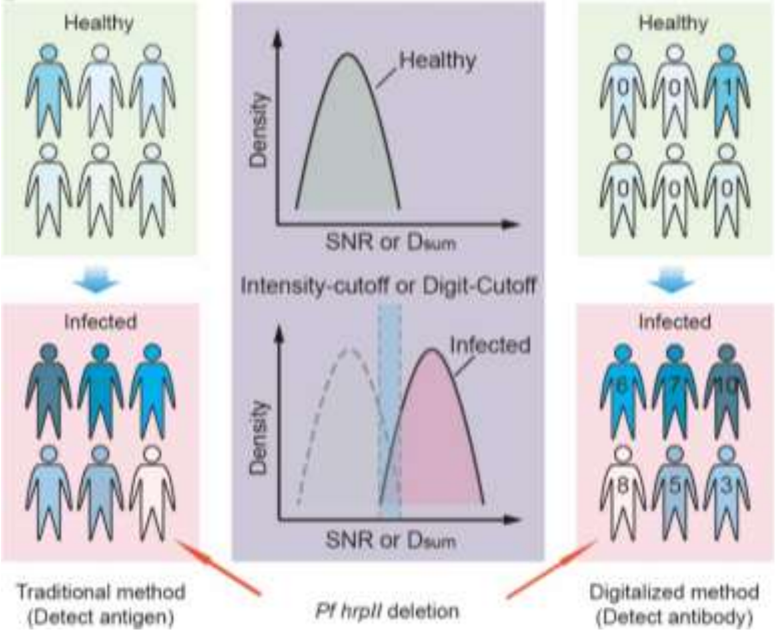

Extended Data Figure 4 Principles of different assays. Although both methods comply with the same general rule of cutoff definition (middle), the traditional method (left panel) uses intensity-cutoff and the binary/digital method (right) uses digit-cutoff. The traditional method is intrinsically false-negative to those HRPII deleted infection, while the binary/digital method is able to detect such infection.

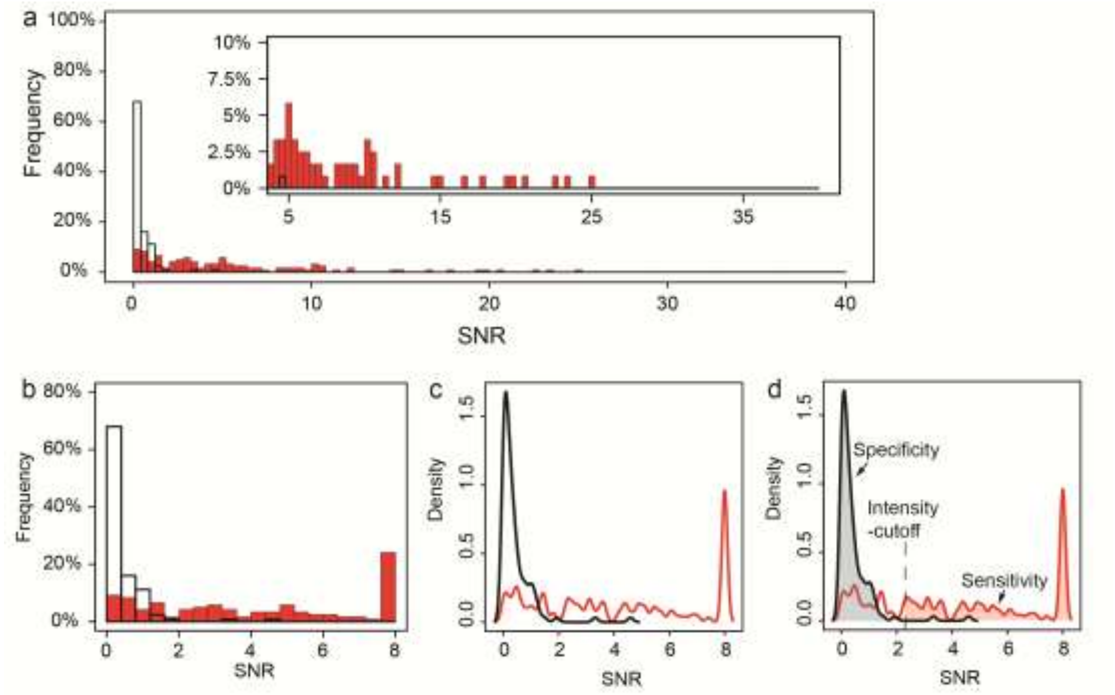

Extended Data Figure 5 Converting SNR vs. Frequency distribution to SNR vs. Density distribution[6, 7]. (a) The SNR vs. frequency plot was constructed from raw data, in which each column stands for the percentage of all sample's response in the corresponding SNR interval of X-axis. Graphic in the range of 3 to 40 of X-axis was amplified as insert image. (b) The SNR that greater than 8 was set to 8. (c) By dividing the frequency by the interval bandwidth, the SNR vs. frequency plot was converted to SNR vs. density plot, which means the whole area under the curve was 1. (d) The areas surrounded by curves and cutoff-line represent the specificity & sensitivity.

Extended Data Table 4 Sensitivity and false positive fraction of 8 ECPs from the training group at different intensity-cutoff

	**SNR = 1**		**SNR = 2**		**SNR = 3**	
	Sens.*	100%-Specificity	Sens.	100%-Specificity	Sens.	100%-Specificity
Pep 1	77.1%	0.0%	72.6%	0.0%	68.2%	0.0%
Pep 2	79.3%	1.6%	74.3%	1.6%	72.6%	0.8%
Pep 3	78.2%	9.6%	68.2%	0.0%	62.6%	0.0%
Pep 4	88.8%	24.8%	78.2%	6.4%	65.4%	2.4%
Pep 5	75.4%	4.8%	65.9%	0.8%	60.3%	0.8%
Pep 6	84.4%	10.4%	75.4%	1.6%	63.1%	1.6%
Pep 7	67.0%	11.2%	55.3%	2.4%	46.4%	0.8%
Pep 8	41.3%	5.6%	30.2%	1.6%	24.0%	1.6%

*Sens.: Sensitivity

For the traditional multiplexing strategy, a sample is judged as a positive sample if any one of the 8 *P. falciparum* peptides has a SNR larger than its individual intensity-cutoff value. Compared with the single index assay, although traditional multiplexing strategy increased the sensitivity from below 80% to 97.2%, an 86.4% specificity disqualified this combination as a *P. falciparum* diagnostic tool. Such contradiction of tuning sensitivity and specificity is commonly observed in multiplexing assay development[8].

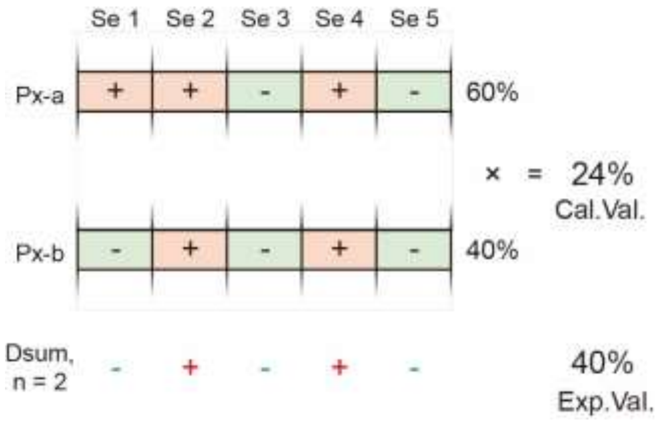

Extended Data Figure 6 Illustration of Cal. Val. and Exp. Val.. Px-a and Px-b represent two of the selected ECPs, Se1~Se5 represent 5 serum samples. "+" symbol in red box represent a positive result of serum-peptide interaction (SNR⩾2)，"-" symbol in blue box represent a negative result of serum-peptide interaction (SNR<2). The coverage of Px-a is 60% (3/5), and of Px-b is 40% (2/5), so the Cal.Val. of these two ECPs is 60%×40%=24%. When use D_{sum} principle, digital cutoff n=2, Se1,3,5 were regarded as negative, and Se2,4 were regarded as positive, so the Exp.Val. of these two ECPs is 40% (2/5). The Exp.Val. usually larger than Cal.Val. when calculating by D_{sum} principle.

For single index (i.e., one peptide), the calculated value is the same as experimental value so they all located along the diagonal line (the open dots in **Fig. 5c** in main text). For any two-peptide combination, we noticed most of the solid red dots are randomly distributed at the upper left side of the diagonal line, indicating any two peptides dominantly present positive correlation. Since the sensitivity of single peptide is < 100%, the sensitivity of any two-peptide combination is smaller than each of the two peptides. The increased overall sensitivity must be attributed to the increase of combinations, from 8 to 28.

Similarly, the specificity of these 28 combinations could be obtained for the healthy samples. Given that our healthy control samples are from areas where *P. falciparum* is a rare infection, the low level of false positive (i.e., 100%-specificity) was due to nonspecific interactions, which are highly possible given the low binary-cutoff of SNR = 2. The combination of such rare and independent events must be even rarer, which is true as indicated by the near zero experimental values (the black dots in **Fig. 5c** in main text). Thus, a simultaneous increase of sensitivity and specificity was achieved.

It is predictable that with the digit-cutoff n increases, the specificity will increase (or saturated at 100%), as shown in **Fig. 5d** for $D_{sum,\ P.\ falciparum} \geq 3$. However, the sensitivity of $D_{sum,\ P.\ falciparum} \geq 3$ decreased to 86%, which was attributed to the fact that the increase of number of combinations (C_8^3, from 28 to 56) does not compensate the decrease of sensitivities of each three-peptide combination. For example, Pep1, 2 and 3 had sensitivity at 72.6%, 74.3% and 68.2%, respectively. The Pep1-2-3 combination gave a calculated value of 0.368 and an experimental value of 0.564. The overall trend of experimental value deviating from the diagonal line may imply intrinsic connections to the immune system but this is not the topic of this study and will be reported elsewhere.

Extended Data Table 5: Homology analysis of 39 *P. falciparum* proteins against *P. viax* proteins.

ID	Query Protein (*P. falciparum*)	Query ID	BLAST Hit Protein (P. vivax)	Hit ID	Query Cover	Ident	MaxScore	TotalScore	E Value
**P01**	HRPII	XP_002808743.1	hypothetical protein	XP_001614388.1	87%	30%	33.1	347	6.00E-05
**P02**	HRPIII	CAX64409.1	hypothetical protein	XP_001616649.1	30%	30%	60.8	164	1.00E-14
**P03**	MSP1	XP_001352170.1	MSP1	ADF48786.1	97%	39%	1010	1138	0.00E+00
**P04**	MSP2	XP_001349578.1	hypothetical protein	XP_001616969.1	85%	22%	44.7	341	3.00E-08
**P05**	MCP1	XP_001347552.1	MCP1	XP_001608496.1	49%	52%	207	207	1.00E-65
**P06**	DBLMSP	XP_001347632.1	nEBP	AHC92543.1	50%	25%	100	117	5.00E-26
**P07**	GLURP	XP_001347628.1	MSP3	ADD39047.1	3%	38%	31.6	31.6	2.00E-04
**P08**	MSP 3	XP_001347629.1	MSP3	XP_001613198.1	37%	36%	50.8	129	7.00E-11
**P09**	MSP 4	XP_001349580.1	MSP4	ADF48689.1	43%	43%	76.6	76.6	2.00E-21
**P10**	MSP 5	XP_001349579.1	MSP5	ACZ55104.1	72%	45%	152	170	2.00E-47
**P11**	MSP 6	XP_001347630.1	MSP3a	XP_001613201.1	22%	49%	48.9	95.5	2.00E-10
**P12**	MSP7.1	XP_001350074.1	MSP7.1,Partial	AEZ00476.1	65%	33%	105	144	1.00E-29
**P13**	MSP7.2	XP_001350075.1	Putative MSP7	ADD39060.1	73%	30%	97.8	117	2.00E-27
**P14**	MSP7.3	XP_002809050.1	MSP7	ACY66915.1	87%	23%	58.5	91.6	2.00E-14
**P15**	MSP7.4	XP_001350079.1	MSP7	ACY66923.1	68%	39%	107	136	4.00E-31
**P16**	MSP7.5	XP_001350080.1	MSP7	XP_001614137.1	62%	37%	138	138	4.00E-41
**P17**	MSP8	XP_001351583.1	MSP8,Partial	AFL93303.1	56%	48%	343	343	3.00E-115
**P18**	MSP9	XP_001350683.1	MSP9	AGR50726.1	79%	33%	243	261	3.00E-72
**P19**	MSP10	XP_966190.1	MSP10	ADV19192.1	41%	57%	211	227	9.00E-66
**P20**	MSP11	XP_001347636.1	hypothetical protein	ADD39021.1	11%	45%	32.7	46.2	8.00E-06
**P21**	EBA-175	XP_001349207.2	nEBP	AHC92543.1	61%	29%	145	340	2.00E-39

Extended Data Table 5 (countinued):

ID	Query Protein (*P. falciparum*)	Query ID	BLAST Hit Protein (*P. vivax*)	Hit ID	Query Cover	Ident	MaxScore	TotalScore	E Value
**P22**	EBA-181	XP_001350957.1	Duffy receptor precursor	XP_001608387.1	46%	31%	124	355	1.00E-32
**P23**	EBA-140	XP_001349859.1	nEBP	AHC92543.1	90%	27%	184	303	5.00E-52
**P24**	EBA-165	XP_001351546.1	DBP Variant 202	AAG53623.1	37%	34%	136	211	4.00E-38
**P25**	AMA-1	XP_001348015.1	AMA-1	ACB42434.1	88%	60%	683	704	0.00E+00
**P26**	PF332	XP_001348162.2	DBSP region II	ACN69890.1	45%	27%	64.7	92.8	7.00E-16
**P27**	RAP1	XP_001348275.1	RAP1	ADH84046.1	99%	39%	510	510	1.00E-173
**P28**	RhopH2	XP_002808967.1	Rhop2	XP_001614833.1	98%	50%	1373	1373	0.00E+00
**P29**	RhopH3	XP_001351928.1	Rhop3	ABR10715.1	95%	55%	1000	1000	0.00E+00
**P30**	CLAG2	XP_001349709.1	CLAG	XP_001616939.1	90%	45%	1117	1117	0.00E+00
**P31**	CLAG3.1	XP_001351100.1	CLAG	XP_001616939.1	96%	44%	1134	1134	0.00E+00
**P32**	CLAG3.2	XP_001351099.1	CLAG	XP_001616939.1	95%	45%	1140	1140	0.00E+00
**P33**	CLAG9	XP_001352222.1	CLAG7	ADV19052.1	99%	53%	1446	1446	0.00E+00
**P34**	CLAG8	XP_002808744.1	CLAG	XP_001614324.1	94%	45%	1098	1098	0.00E+00
**P35**	EMP2/MESE	XP_001351567.1	hypothetical protein	XP_001615965.1	15%	52%	79	219	5.00E-18
**P36**	Serine repeat antigen 5	XP_001349586.1	Serine repeat antigen	XP_001612999.1	94%	44%	665	766	0.00E+00

Extended Data Table 6 Sensitivity, specificity, negative and positive predictive values and AUC of 8 ECPs in detecting training group.

peptide	Sensitivity (95% CI)	Specificity (95% CI)	PPV* (95%CI)	NPV** (95%CI)	AUC (95% CI)
**Pep 1**	0.69(0.62,0.76)	1.00(0.96,1.00)	1.00(0.96,1.00)	0.69(0.62,0.76)	0.87(0.83,0.92)
**Pep 2**	0.73(0.66,0.80)	0.98(0.94,1.00)	0.98(0.95,1.00)	0.72(0.65,0.79)	0.90(0.86,0.93)
**Pep 3**	0.68(0.61,0.75	0.99(0.96,1.00)	0.99(0.96,1.00)	0.69(0.61,0.75)	0.86(0.82,0.90)
**Pep 4**	0.80(0.73,0.85)	0.94(0.88,0.97)	0.95(0.90,0.98)	0.76(0.69,0.83)	0.92(0.88,0.95)
**Pep 5**	0.66(0.59,0.73)	0.99(0.96,1.00)	0.99(0.95,1.00)	0.67(0.60,0.74)	0.88(0.84,0.92)
**Pep 6**	0.75(0.68,0.82)	0.98(0.94,1.00)	0.99(0.95,1.00)	0.74(0.66,0.80)	0.93(0.90,0.96)
**Pep 7**	0.55(0.48,0.63)	0.97(0.92,0.99)	0.96(0.90,0.99)	0.60(0.53,0.67)	0.87(0.83,0.91)
**Pep 8**	0.30(0.24,0.37)	0.98(0.94,1.00)	0.96(0.88,1.00)	0.50(0.43,0.56)	0.83(0.78,0.87)
**Total**	0.97(0.94,0.99)	0.86(0.78,0.91)	0.91(0.86,0.94)	0.96(0.90,0.99)	0.98(0.97,1.00)

*PPV: positive predictive values
**NPV: negative predictive values

Extended Data Table 7 Validation of the statistical correlation.

		P. falciparum	Healthy	Sensitivity (95% CI)	Specificity (95% CI)	PPV (95%CI)	NPV (95%CI)	kappa index (P value*)
**Training group**	Positive	166	2	0.93(0.88,0.96)	0.98(0.94,1.00)	0.99(0.96,1.00)	0.90(0.84,0.95)	0.90(<0.01)
	Negative	13	123					
**Test group**	Positive	231	9	0.95(0.91,0.97)	0.99(0.98,1.00)	0.96(0.93,0.98)	0.99(0.98,0.99)	0.94(<0.01)
	Negative	13	1034					

*U test

1. Zhang, Y. et al. Proteomic Analysis of Plasmodium falciparum Schizonts Reveals Heparin-Binding Merozoite Proteins. *Journal of Proteome Research* **12**, 2185-2193 (2013).
2. White, A.M., Daly, D.S., Willse, A.R., Protic, M. & Chandler, D.P. Automated Microarray Image Analysis Toolbox for MATLAB. *Bioinformatics* **21**, 3578-3579 (2005).
3. Tusher, V.G., Tibshirani, R. & Chu, G. Significance analysis of microarrays applied to the ionizing radiation response. *Proc Natl Acad Sci U S A* **98**, 5116-5121 (2001).
4. Edward A. Sasse, P.D. et al. How to Define and Determine Reference Intervals in the Clinical Laboratory; Approved Guideline, Vol. **20**, Edn. Second Edition. (NCCLS, USA; 2000).
5. Tao, Z.Y. et al. Congenital Malaria in China. *PLoS Negl Trop Dis* **8**, e2622 (2014).
6. SCOTT, D.W. Multivariate Density Estimation: Theory, Practice, and Visualization. (JOHN WlLEY & SONS, INC., USA; 1992).
7. Venables, W.N. & Ripley, B.D. Modern Applied Statistics With S, Edn. 4th. (springer, USA; 2002).
8. Chapman, C.J. et al. Autoantibodies in lung cancer: possibilities for early detection and subsequent cure. *Thorax* **63**, 228-233 (2008).
